# Successful treatment of diffuse cutaneous leishmaniasis caused by *Leishmania amazonensis*^☆^^☆☆^

**DOI:** 10.1016/j.abd.2021.03.003

**Published:** 2021-07-15

**Authors:** Raimunda Nonata Ribeiro Sampaio, Marina Freitas Ferreira, Sofia Sales Martins, Jorgeth de Oliveira Carneiro da Motta

**Affiliations:** Hospital Universitário de Brasília, Universidade de Brasília, Brasília, DF, Brazil

**Keywords:** Allopurinol, Antimony, Recurrence, Therapy, Diffuse cutaneous leishmaniasis

## Abstract

Diffuse cutaneous leishmaniasis is a rare universal disease associated with an inadequate host cell immune response, caused by different species: *infantum*, *aethiopica, major*, *mexicana,* and others, which presents the challenge of a poor therapeutic response. In Brazil, it is caused by *L. amazonensis*. A case confirmed by histopathology with an abundance of vacuolated macrophages full of amastigotes and lymphocyte scarcity, identified by RFLP-ITS1PCR and *in vitro* decrease and exhaustion of the host cell immune response to *L. amazonensis* antigen, was treated early (3 months after the onset) with Glucantime (2 months) and allopurinol (29 months) with clinical cure, after a follow-up for 30 months after treatment.

Diffuse cutaneous leishmaniasis (DCL) is a rare universal disease associated with an inadequate host cell immune response, caused by different species: *infantum*, *aethiopica, major*, *mexicana* and others. In Brazil, it is caused by *L. amazonensis*, perhaps a subspecies that, upon failure of the host's cell response, replicates uncontrollably, resulting in disease severity and chronicity.

In its initial form, it usually presents as a slow-growing erythematous macula or plaque, simulating diseases such as lupus vulgaris, sarcoidosis and others. Subsequently, after an average of 3 years, it spreads with the formation of plaques, usually non-ulcerated nodules and not affecting the mucosa, characterizing the best-known picture of diffuse cutaneous leishmaniasis by *Leishmania (L) amazonensis*.

Its treatment represents a challenge due to constant recurrences, but the knowledge related to its treatment is limited to clinical cases. Initially, it was performed with conventional monotherapy.^1^, ^2^ More recently, the time of treatment with monotherapy has been prolonged and a combination of drugs has also been used, both with failure reports.^3^, ^4^ This is the report of a case of DCL diagnosed early and successfully treated with N-methyl glucamine antimoniate (NMG) associated with allopurinol.

A 65-year-old male patient, born in the state of Minas Gerais, had an erythematous plaque measuring 4 × 2 cm on the dorsum of the nose (Fig. 1), which had appeared 2 months after going fishing in the state of Amazonas. The smear showed abundant parasites, fast-growing culture, and the identification of *L. amazonensis* (RFLP-ITS1PCR). Montenegro's intradermoreaction and indirect immunofluorescence were negative. Histopathological analysis showed an abundance of vacuolated macrophages full of leishmania and lymphocyte scarcity (Fig. 2). The *in vitro* assay of the patient's peripheral blood showed decrease and exhaustion of the host’s cell immune response to the *L. amazonensis* antigen, detected by flow cytometry. After 3 months of evolution, the patient was initially treated with NMG 20 mgSbV/kg/day for 20 days without improvement. Soon, the antimony was reintroduced associated with 1,200 mg of allopurinol/day for 2 months (cumulative dose of 44,625 mgSbV) and the lesion regressed to mild infiltration at the time when the antimony was suspended while allopurinol was maintained for 29 months with gradual dose reductions. Thirty months after the end of the treatment, the patient had only a dyschromic atrophic scar (Fig. 3).

**Figure 1:**
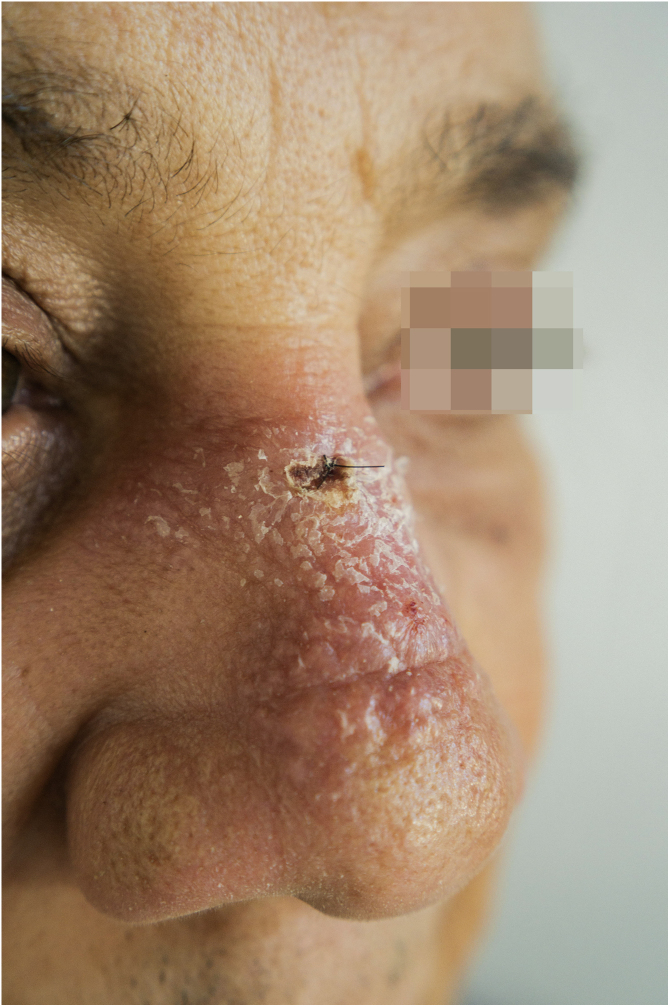
Erythematous, scaling plaque on the nose.

**Figure 2:**
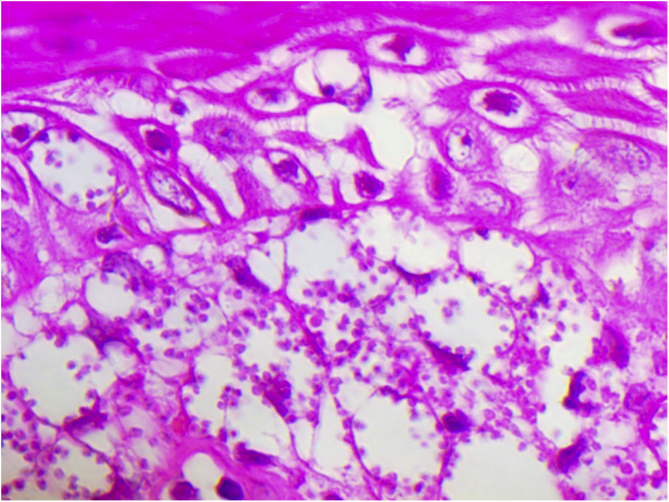
Vacuolated macrophages containing abundant amastigotes (Hematoxylin & eosin, ×400).

**Figure 3:**
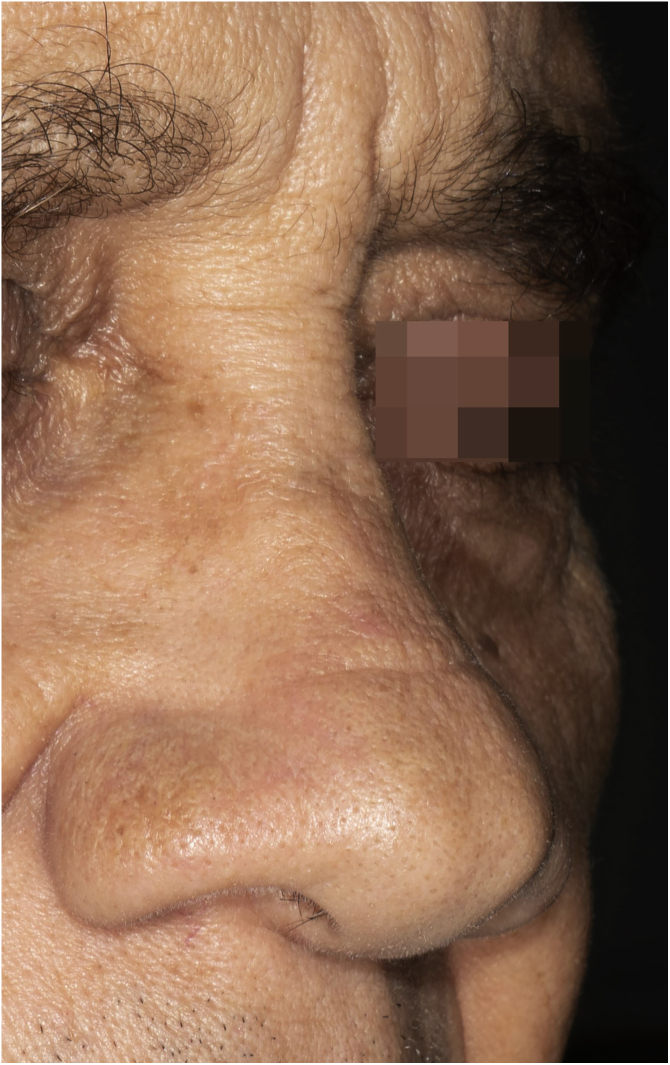
Atrophic and dyschromic scar on the dorsum of the nose.

Both the diagnosis and treatment were early. The time-to-cure ratio in American tegumentary leishmaniasis (ATL) is believed to be inversely proportional.^5^ In DCL cases, there seems to be a PD-L1-mediated T lymphocyte depletion leading to low cytotoxicity and low IFNy production in response to the leishmania antigen *in vitro*.^6^, ^7^ On the other hand, the antimony destroys the parasites through direct action and indirectly increases the phagocytosis of monocytes and neutrophils and the superoxide anion. Allopurinol, in turn, is a leishmanicidal and immunomodulator.^8^

The strategy to improve the effectiveness of antimony using an immunomodulator is promising, according to some researchers.^9^ Also, targeted therapy involving the PD-1/PDL-1 pathway was effective in reducing the parasite load in a murine model and constitutes a hopeful strategy after the failure of traditional drugs.^10^ Finally, it is worth asking to what extent, the early and prolonged treatment may have influenced the therapeutic outcome of this case of DCL and whether they may indicate perspectives for a successful future treatment.

## Financial support

FUNADERM (Notice 2016); Fundação de Apoio à Pesquisa – Distrito Federal – FAP-DF(Project N. 0193.001447/2016).

## Authors’ contributions

Raimunda Nonata Ribeiro Sampaio: Conception and design; analysis and interpretation of the data; writing; critical review and final review.

Marina Freitas Ferreira: Collection of clinical data from the medical records; literature review; interpretation; first draft.

Sofia Sales Martins: Laboratory data collection; data interpretation and review.

Jorgeth de Oliveira Carneiro da Motta: Data collection and clinical follow-up; analysis; interpretation and review of data.

## Conflicts of interest

None declared.
